# Estimated Habitual Dairy Polar Lipid Exposure and Post-Intervention Plasma Lipid Outcomes in Perimenopausal Women in Latvia: A Secondary Exposure-Based Analysis of a 28-Day Fermented Buttermilk Randomised Trial

**DOI:** 10.3390/nu18132194

**Published:** 2026-07-06

**Authors:** Svetlana Aleksejeva, Vitalijs Radenkovs, Maksims Zolovs, Ilona Vilkoite, Laila Meija, Inga Ciprovica

**Affiliations:** 1 Faculty of Agriculture and Food Technology, Latvia University of Life Sciences and Technologies, LV-3004 Jelgava, Latvia; inga.ciprovica@lbtu.lv; 2 Division of Smart Technologies, Latvia University of Life Sciences and Technologies, LV-3001 Jelgava, Latvia; vitalijs.radenkovs@lbtu.lv; 3 Institute of Horticulture, LV-3701 Dobele Municipality, Latvia; 4 Statistics Unit, Rīga Stradiņš University, LV-1067 Riga, Latvia; maksims.zolovs@du.lv; 5 Institute of Life Sciences and Technology, Daugavpils University, LV-5401 Daugavpils, Latvia; 6 Department of Doctoral Studies, Rīga Stradiņš University, LV-1067 Riga, Latvia; ilona153@inbox.lv; 7 Department of Rehabilitation, Rīga Stradiņš University, LV-1067 Riga, Latvia; laila.meija@rsu.lv

**Keywords:** dairy polar lipids, phosphatidylcholine, phosphatidylethanolamine, sphingomyelin, perimenopause, buttermilk, FFQ, dietary exposure estimation, seasonal variation, HDL-C

## Abstract

**Background/Objectives:** Hormonal changes during the perimenopausal period are linked to alterations in lipid metabolism and increased cardiovascular disease risk. Dairy polar lipids (PLs), including phosphatidylcholine (PC), phosphatidylethanolamine (PE), and sphingomyelin (SM), have attracted growing interest for their potential effects on intestinal cholesterol absorption, lipoprotein metabolism, and circulating lipid concentrations. However, evidence on habitual dietary exposure to naturally occurring dairy PLs remains limited. The aim of the present study was to estimate habitual dietary exposure to dairy PLs by integrating season-specific dairy product composition with individual dietary intake data, and to examine whether this estimated exposure was associated with post-intervention plasma lipid outcomes in perimenopausal women in Latvia. **Methods:** The analysis included 61 perimenopausal women, comprising an intervention group (*n* = 31) and a control group (*n* = 30), who were further stratified into winter and spring cohorts. The intervention group consumed 250 mL day^−1^ of fermented buttermilk for 28 days. Dietary intake was assessed using a food frequency questionnaire (FFQ). Season-specific concentrations of total PL, PC, PE, and SM were quantified in locally produced dairy products using LC-ESI-MRM-TQ-MS/MS and integrated with individual dietary intake data to estimate dairy PL exposure. Multivariable linear regression models were adjusted for baseline lipid concentrations, intervention group, season, and total dairy PL intake. **Results:** Estimated total dairy PL intake was positively associated with post-intervention HDL-C concentrations in the adjusted models (β = 0.059; 95% CI: 0.020–0.099), whereas no statistically significant associations were observed for total cholesterol (TC), low-density lipoprotein cholesterol (LDL-C), or triglycerides (TGs). After adjustment for baseline lipid concentrations, season, and estimated total dairy PL intake, intervention-group allocation was associated with higher post-intervention TC (β = 0.485; 95% CI: 0.023–0.948) and LDL-C (β = 0.330; 95% CI: 0.024–0.636) than in the control group. The spring-versus-winter season indicator was not independently associated with lipid outcomes. **Conclusions:** Estimated habitual dairy PL exposure was positively associated with post-intervention HDL-C concentrations but not with TC, LDL-C, or TGs. These findings do not support a clear lipid-lowering effect of the 28-day fermented buttermilk intervention in this secondary, exposure-based analysis. The results should be interpreted with caution, as dairy PL exposure was estimated from FFQ-derived intake data and season-specific product composition rather than measured directly. This trial was retrospectively registered in the ISRCTN registry ISRCTN11974930, registered on 25 June 2026.

## 1. Introduction

The menopausal transition is a clinically relevant period for cardiometabolic risk assessment, as declining estrogen concentrations are linked to adverse changes in lipid metabolism in women. During perimenopause, increases in total cholesterol (TC), low-density lipoprotein cholesterol (LDL-C), and triglycerides (TGs), together with changes in high-density lipoprotein cholesterol (HDL-C), may contribute to a more atherogenic lipid profile and a higher risk of cardiovascular disease (CVD). Identifying dietary factors that may support lipid metabolism during this life stage is therefore relevant for nutrition-based prevention strategies [1,2,3,4,5].

In dairy products, polar lipids (PL) are primarily associated with the milk fat globule membrane (MFGM), making dietary exposure dependent on both milk composition and processing [6]. Major dairy PL classes include phosphatidylcholine (PC), phosphatidylethanolamine (PE), and sphingomyelin (SM), which contribute to membrane structure, lipid transport, and cellular signalling [7,8]. Dairy PLs have been suggested to influence plasma lipid metabolism, although evidence for a direct role in CVD risk reduction remains limited [9,10,11,12]. However, these findings are derived mainly from controlled interventions using isolated or enriched PL sources rather than habitual exposure to naturally occurring dairy PLs [13,14,15]. In addition, human and experimental studies suggest that milk PLs may influence cholesterol homeostasis, lipid bioavailability, and related metabolic pathways, but the translation to real-world dietary exposure remains insufficiently characterised [14,15,16].

In perimenopausal women, lipid metabolism is influenced by hormonal transition and cardiometabolic vulnerability; therefore, differences in habitual exposure to bioactive dairy lipid fractions may be relevant when examining lipid-related outcomes. Seasonal variation in dairy product consumption has been associated with changes in raw milk composition and membrane-associated lipid fractions, influencing the concentration and distribution of milk PL classes, including PC, PE, and SM [17,18,19,20,21,22,23].

These seasonal differences are relevant to exposure estimation because the same reported dairy intake may correspond to different PL exposure levels, depending on the season-specific PL composition of the products consumed. In the present study, season was considered primarily as a factor influencing estimated dairy PL exposure, rather than as an independent determinant of lipid response.

Further processing alters dairy PL exposure by redistributing MFGM-associated lipids across dairy fractions. Separation, cream churning, and buttermilk release can lead to selective enrichment or depletion of PLs in different dairy products [14,16,24,25].

Therefore, consumer exposure to dairy PLs cannot be inferred solely from raw milk composition; it depends on the PL concentration in the specific dairy products consumed and on their relative contributions to habitual dietary intake.

Although milk PLs have been studied in isolated fractions and enriched formulations, less is known about real-life dietary exposure to naturally occurring dairy PLs. Most available human evidence derives from controlled intervention settings using enriched ingredients, supplements, or specific dairy matrices. In contrast, habitual exposure to naturally occurring dairy PLs from ordinary dairy products remains poorly characterised, particularly when individual dietary intake, product-specific PL composition, seasonal variation, and plasma lipid outcomes are considered together. Therefore, the present analysis addresses a distinct knowledge gap: it estimates participant-level habitual dairy PL exposure from FFQ-derived dairy intake and experimentally measured season-specific dairy product composition and evaluates whether this estimated exposure is associated with post-intervention lipid outcomes. Season-specific PL composition and processing-related redistribution have rarely been integrated with individual dietary intake data to evaluate associations with plasma lipid outcomes in human populations. This gap is relevant because habitual dairy intake may expose individuals to varying amounts and PL classes, depending on season and dairy product.

The present analysis extends a previously published 28-day fermented buttermilk intervention conducted among perimenopausal women in Latvia [12]. In contrast to the original intervention report, this exposure-based analysis integrates experimentally quantified, season-specific PL composition of dairy products with dietary intake data to estimate dietary exposure to dairy PLs.

The aim of the present study was to estimate habitual dietary exposure to dairy PLs by integrating season-specific dairy product composition with individual dietary intake data, and to examine whether this estimated exposure was associated with post-intervention plasma lipid outcomes among perimenopausal women in Latvia.

## 2. Materials and Methods

### 2.1. Patient and Public Involvement

Participants and members of the public were not involved in setting the research question, designing the trial, selecting outcomes, interpreting the results, or preparing the manuscript. Women enrolled in the trial participated only as study participants.

### 2.2. Study Design and Participants

The original study was a 28-day, two-arm, parallel-group, randomised controlled dietary intervention trial conducted in Latvia among free-living perimenopausal women. Participants were allocated in an approximately 1:1 ratio to either the fermented buttermilk intervention group or the control group. The original trial evaluated lipid outcomes after daily consumption of fermented buttermilk. The present manuscript reports a secondary, exploratory exposure-based analysis of this completed trial by integrating participant-level dietary data with season-specific dairy PL composition data to estimate dairy PL exposure and evaluate its association with post-intervention lipid profile parameters.

Participant recruitment, baseline assessment, intervention delivery, and post-intervention follow-up were conducted from October 2023 to June 2024. Each participant completed a 28-day trial period, with lipid profile measurements performed at baseline and after completion of the intervention period.

The present analysis was restricted to winter and spring because these were the only seasons for which complete participant-level dietary assessments, blood lipid measurements, and matched dairy product PL composition data were available from the original trial. Winter and spring, therefore, represent the available clinical and analytical cohorts rather than a full four-season sampling design. Accordingly, the seasonal variable in the regression models should be interpreted as a spring-versus-winter indicator rather than as a measure of full annual seasonal variation.

A total of 61 perimenopausal women were included in the present analysis. Participants were assessed in two seasonal cohorts: winter (*n* = 23) and spring (*n* = 38). The intervention group comprised 31 participants, and the control group 30. Eligible participants were women aged 45–55 years residing in Latvia with perimenopausal symptoms and moderately elevated low-density lipoprotein cholesterol (LDL-C) concentrations (3.01–4.12 mmol L^−1^) over the previous 6 months. Key exclusion criteria included primary hyperlipidemia, established cardiovascular disease, diabetes mellitus, thyroid or adrenal disease, liver or kidney disease, history of gastrointestinal surgery, hormonal therapy, immunosuppressive therapy, and lipid-lowering or antihypertensive treatment. Detailed recruitment procedures, allocation, and baseline participant characteristics have been reported previously [12].

The planned sample size was based on feasibility considerations for the original clinical trial and the resources available for participant recruitment, intervention delivery, laboratory testing, and follow-up. The target sample size was approximately 30 participants per group, corresponding to a minimum total sample size of approximately 60 participants.

Participants were randomised using restricted block randomisation to support balanced allocation between the intervention and control groups during recruitment. The random allocation sequence was generated before group allocation by the principal investigator using Microsoft Excel. As participants entered the study, they were assigned sequentially to the next available allocation in the randomisation list, with each block completed before moving to the next. No additional allocation concealment procedure was documented in the available study records. The researcher enrolling participants and assigning them to the intervention or control group had access to the allocation sequence at the time of assignment.

All eligible participants then entered a 28-day trial period. Participants in the intervention group were instructed to consume 250 mL day^−1^ of commercially available fermented buttermilk with 0.5% fat (Baltais, AS Tukuma piens, Tukums, Latvia) for 28 consecutive days. They were asked not to make any additional dietary or lifestyle changes during the intervention period. The buttermilk was delivered to participants by the researcher or a courier to ensure it remained fresh. Participants in the control group were instructed to maintain their usual diet and lifestyle throughout the same period.

During the trial, participants completed 24 h and 72 h food diaries over three consecutive days. The researcher provided guidance, including a video tutorial and a question-and-answer session. Dietary records were completed using an atlas of food products and portion sizes from the Institute of Food Safety, Animal Health and Environment “BIOR”. At the end of the 28-day trial, post-intervention blood tests were conducted in both groups to measure TC, LDL-C, HDL-C, and TGs. In total, 61 women completed the study, with 31 in the intervention group and 30 in the control group.

Because this was a pragmatic, food-based intervention using a commercially available fermented dairy product, participant blinding was not feasible. Participants, the researcher responsible for enrollment and group allocation, and the data analyst were not blinded to group allocation. Biochemical measurements were performed by an accredited clinical laboratory according to standard operating procedures.

Participants in both groups were instructed to maintain their habitual diet and lifestyle during the 28-day trial period and to report any relevant changes in medication, supplement use, diet, or health status.

### 2.3. Reagents and Standards

High-purity reagents and liquid chromatography-mass spectrometry (LC-MS)-grade solvents were used for PL extraction and analysis. LC-MS-grade acetonitrile (MeCN), 2-propanol (isopropanol, IPA), and formic acid were purchased from Sigma-Aldrich (Darmstadt, Germany). Ammonium formate (HCOONH4, purity ≥99.0%) was obtained from Merck KGaA (Darmstadt, Germany). Chloroform (CHCl3, purity ≥99.8%), methanol (CH3OH, purity ≥99.9%), and LC-MS-grade water were purchased from VWR International (Paris, France).

Phospholipid analytical standards, including SM(d18:1/16:0), SM(d18:1/18:0), PE(16:0/18:1), PE(16:0/18:2), PC(16:0/18:1), and PC(18:1/18:1), were obtained from Sigma-Aldrich (St. Louis, MO, USA). All phospholipid standards were supplied as mixtures representative of each lipid class. Individual molecular species within each class-standard mixture were characterised and monitored using their specific MRM transitions and retention times. These class-standard mixtures were used to prepare external calibration curves over the concentration range of 0.0175–0.56 µg mL^−1^, with six calibration levels injected in triplicate.

Quantification was performed using external calibration with commercially available PL standards for the selected PL species or lipid classes. Individual PL classes were identified using predefined multiple reaction monitoring (MRM) transitions and quantified against the corresponding calibration curves. Because class-based standards may differ in their response factors across molecular species, this was taken into account when interpreting absolute concentrations.

### 2.4. Ethics Statement

The study was conducted in accordance with the Declaration of Helsinki and approved by the Ethics Committee of Rīga Stradiņš University (protocol code 2-PĒK-4/513/2022; approval date: 23 November 2022). All participants provided written informed consent prior to participation.

### 2.5. Trial Registration, Protocol, and Statistical Analysis Plan

This trial was retrospectively registered in the ISRCTN registry ISRCTN11974930, registered on 25 June 2026; https://www.isrctn.com/ISRCTN11974930 (accessed on 25 June 2026). Prospective trial registration was not performed before participant enrolment because the study was initiated as an investigator-initiated, food-based dietary intervention conducted within a doctoral research project, and prospective public trial registration was not required as part of the institutional ethics approval or local study initiation process at that time. Following the journal’s recommendation, the trial was retrospectively registered with the ISRCTN registry to ensure transparent public documentation of the study. The study protocol, eligibility criteria, intervention, and prespecified primary outcomes were established before participant enrolment and remained unchanged throughout the trial. The study protocol was approved by the Ethics Committee of Rīga Stradiņš University before participant recruitment. A separate publicly accessible statistical analysis plan was not prepared before trial initiation. No changes to the original trial intervention, comparator, eligibility criteria, or lipid outcome measurements were made after trial commencement. The present secondary exposure-based analysis was developed after completion of the original 28-day intervention and after the availability of season-specific dairy polar lipid composition data.

### 2.6. Dietary Assessment and Estimation of Dairy Intake

Dietary intake was assessed using a semi-quantitative food frequency questionnaire (FFQ) adapted from the Latvian National Dietary Survey [26]. The FFQ captured habitual dietary intake over the preceding 12 months and included approximately 260 food items grouped into major food categories. The FFQ was completed with the researcher’s assistance during online interviews. To minimise misclassification of similar food products, participants received clarification on product categories and portion sizes as needed. Dietary assessment was further supported by 24 h and 72 h food diaries completed with guidance, including a video tutorial, question-and-answer sessions, and the food product and portion atlas. These records were used to verify the consistency of reported food frequencies and quantities.

PL exposure was based on FFQ-derived average daily intake values. Portion sizes were estimated using the BIOR’s standardised food product and portion atlas of the Institute of Food Safety, Animal Health and Environment [27]. Nutritional data were processed in Microsoft Dynamics AX 2009.

Participants were assigned to winter or spring cohorts based on the timing of dietary assessment and blood sampling. FFQ-derived habitual dairy intake was combined with season-specific PL composition data to estimate each participant’s dietary dairy PL exposure. Average daily dairy intake was standardised and expressed as milk equivalents using predefined conversion factors [28,29]. Average daily intake of milk and dairy products expressed as milk equivalents during winter and spring is provided in Supplementary Table S2. Plant-based dairy substitutes were excluded from dairy PL exposure calculations.

### 2.7. Blood Lipid Profile Assessment

Blood samples were collected at baseline and at the end of the 28-day intervention period. Lipid profile measurements included total cholesterol (TC), low-density lipoprotein cholesterol (LDL-C), high-density lipoprotein cholesterol (HDL-C), and triglycerides (TGs). Blood analyses were performed at the accredited clinical laboratory, Centrālā Laboratorija Ltd. (Rīga, Latvia; accreditation certificate LATAK-M-434-04-2011). Lipid profile parameters were measured using the enzymatic colourimetric method in accordance with the laboratory’s standard operating procedures.

### 2.8. Outcomes

The primary lipid outcomes were post-intervention TC, LDL-C, and HDL-C concentrations after the 28-day intervention period. TGs were analysed as an additional secondary lipid outcome. For each lipid parameter, the main analysis metric was the post-intervention concentration adjusted for the corresponding baseline lipid concentration. Baseline and post-intervention lipid concentrations were measured in mmol L^−1^. Change-from-baseline values are presented descriptively in the Supplementary Materials.

### 2.9. Harms and Adverse Events

Potential harms and unintended events were assessed non-systematically through participant self-report during the 28-day trial period. Participants were asked to report any difficulties with fermented buttermilk consumption, non-compliance, changes in health status, or adverse events during the intervention period.

### 2.10. Dairy Product Sampling

Raw milk was obtained from a dairy producer (Latvijas Piens Ltd., Jelgava, Latvia) in winter (December 2023) and spring (May 2024). These sampling periods were selected to align with the available clinical assessment windows of the original trial. Seasons were defined by the sampling period and reflected local seasonal conditions in Latvia. For each season, raw milk was processed on a laboratory scale to produce skimmed milk, cream, and buttermilk by churning. Raw milk, skimmed milk, cream, and buttermilk were included in the PL compositional dataset.

The chemical composition of raw milk was determined using a milk analyser (MilkoScan^TM^ Mars; Foss, Hillerød, Denmark). Raw milk was heated to 40 °C and then separated using a laboratory-scale separator (Armfield, Hampshire, UK) to obtain skimmed milk and cream. The chemical composition of skimmed milk and cream was measured using the corresponding MilkoScan^TM^ Mars calibration tools. The separated cream was pasteurised at 90 °C for 5 min, cooled, and matured at 6 °C for 6 h. Cream maturation was performed at 6 ± 2 °C. After maturation, the cream was heated to 12 °C and churned using a laboratory-scale butter churn FJ 10 (Janschitz, Althofen, Austria) at 11–12 °C until butter grains formed and buttermilk was released. The buttermilk was drained, and the butter grains were further processed into a homogeneous butter mass. The composition of the buttermilk was analysed using the MilkoScan^TM^ Mars milk analyser.

Processing and corresponding compositional measurements were conducted in triplicate for each season. Replicate results were summarised at the product-season level to estimate dietary exposure.

Raw milk, skimmed milk, cream, and buttermilk, as well as commercial fermented buttermilk used during the intervention period, were analysed for total PL concentration and selected major PL classes, including PC, PE, and SM. The relative proportions of PC(16:0/18:1), PC(18:1/18:1), PE(16:0/18:2), PE(16:0/18:1), SM(d18:1/16:0), and SM(d18:1/18:0) were used to characterise PL patterns and seasonal variation across the products studied.

### 2.11. Intervention Product Characterisation

The intervention product was commercially available fermented buttermilk (Baltais, AS Tukuma piens, Tukums, Latvia), provided to participants in the intervention group at a dose of 250 mL day^−1^ for 28 days. Product samples from the winter and spring intervention periods were analysed for total concentrations of PL, PC, PE, and SM using the same LC-ESI-MRM-TQ-MS/MS method described below. To improve exposure interpretation, PL concentrations, expressed as mg 100 g^−1^, were recalculated to the daily intervention portion of 250 mL, assuming that 250 mL corresponded to approximately 250 g of product.

### 2.12. PL Extraction and LC-ESI-MRM-TQ-MS/MS Analysis

PLs were extracted from homogenised dairy product samples using a modified Bligh and Dyer protocol optimised for LC–electrospray ionisation–MRM–triple quadrupole tandem mass spectrometry (LC-ESI-MRM-TQ-MS/MS). Briefly, 1.0 g of homogenised dairy product sample was transferred to a 15 mL centrifuge tube, followed by the addition of 3.0 mL of chloroform and 6.0 mL of methanol. The mixture was vortexed for 1 min and sonicated for 15 min at 20 ± 1 °C. Phase separation was achieved by adding 3.0 mL of water, vortexing for 30 s, and centrifuging at 9280× *g* for 10 min at 4 °C. The lower chloroform phase containing lipids was collected, dried under a gentle stream of nitrogen to dryness, and freeze-dried for 24 h to remove residual water. The dried extract was reconstituted in 3.0 mL of IPA:MeOH (50:50, *v*/*v*).

Before LC-MS/MS analysis, samples were vortexed and ultrasonicated for 5 min to ensure complete solubilisation. Extracts were diluted with the initial mobile phase (60% water and 40% MeCN) to bring analyte concentrations within the calibration range. All extracts were analysed within 12 h of preparation. The PL concentration was determined using an LC-ESI-MRM-TQ-MS/MS method. Chromatographic separation was performed on a Discovery HS F5-3 column (3.0 µm, 150 × 2.1 mm; Merck KGaA) at 40 °C. Mobile phase A consisted of water containing 10 mM ammonium formate and 0.1% formic acid. Gradient elution was performed at a flow rate of 0.25 mL min^−1^, with an injection volume of 3 µL. Detection was performed in positive electrospray ionisation mode using predefined MRM transitions for PC(16:0/18:1), PC(18:1/18:1), PE(16:0/18:2), PE(16:0/18:1), SM(d18:1/16:0), and SM(d18:1/18:0). The MRM transitions and representative retention times for the quantified lipid species are shown in Supplementary Table S5. Representative extracted-ion chromatograms of the target lipid standards are provided in Supplementary Figure S1. The chromatograms show MRM traces and retention times for SM(d18:1/16:0), SM(d18:1/18:0), PE(16:0/18:1), PE(16:0/18:2), PC(16:0/18:1), and PC(18:1/18:1), demonstrating the chromatographic performance of the method. Data acquisition and processing were performed using LabSolutions Insight LC-MS software (version 3.7 SP3).

External calibration curves were prepared using phospholipid standards over a concentration range of 0.0175–0.56 µg mL^−1^. Six calibration levels were injected in triplicate. The calibration curves showed acceptable linearity for all quantified PL classes (R^2^ > 0.995), and analytical precision, expressed as the relative standard deviation (SD), was ≤15%. Total PL, PC, PE, and SM concentrations were reported as mg 100 g^−1^ of dairy product. The relative proportions of PC, PE, and SM were reported as percentages of the quantified PL classes.

The lipid annotations reported in this study correspond to targeted molecular species rather than to lipid headgroup classes alone. The quantified species were PC(16:0/18:1), PC(18:1/18:1), PE(16:0/18:2), PE(16:0/18:1), SM(d18:1/16:0), and SM(d18:1/18:0), identified using predefined precursor-to-product ion transitions and retention times. However, the method does not determine fatty-acyl sn-positions or fully resolve all possible isobaric or isomeric species. Therefore, these annotations should be interpreted as targeted LC-MRM-MS/MS molecular-species assignments, not as complete structural elucidation.

### 2.13. Data Processing and Exposure Preparation

FFQ-derived dairy intake data were processed to obtain average daily intake values. Dairy product intake was expressed in milk equivalents using predefined conversion factors.

The exposure variable should be interpreted as an estimate of habitual dairy PL exposure based on the participant’s reported usual dairy intake, not as a direct measure of PL intake during the 28-day intervention period. Season-specific dairy PL composition values were applied according to the participant’s clinical assessment season to reflect the dairy product composition available during the corresponding sampling window. This approach approximates season-matched habitual exposure, but it cannot fully capture within-person seasonal changes in dairy intake across the entire year.

The estimated daily intake of total quantified dairy PL, PC, PE, and SM was calculated by combining FFQ-derived dairy intake with season-specific PL concentrations measured in dairy products. For each participant, the intake of each dairy product category, expressed in milk equivalents, was multiplied by the corresponding season-specific concentration of total quantified PL, PC, PE, or SM. PL concentrations were expressed as mg 100 g^−1^; the resulting values were divided by 100 to obtain the estimated intake in mg day^−1^.

Total quantified dairy PL intake was defined as the sum of the quantified PC, PE, and SM species measured by the targeted LC-ESI-MRM-TQ-MS/MS method. It should not be interpreted as representing the complete composition of dairy PLs.

For regression analyses, total quantified dairy PL intake was log-transformed, as visual inspection indicated right-skewness and potential influence from extreme values. For exploratory PCA, relative PL class composition data were standardised using Z-score normalisation. PC/PE ratios and ternary compositional plots were generated to visualise the relative balance of PC, PE, and SM across dairy products and seasons.

### 2.14. Statistical Analysis

To evaluate associations between estimated dairy PL exposure and lipid profile parameters, separate ordinary least-squares regression models were fitted for each outcome. Post-intervention lipid concentrations were used as the dependent variables. The corresponding baseline lipid concentration, intervention group, season, and log-transformed total quantified dairy PL intake were included as independent variables. TGs were analysed as an additional secondary lipid outcome using the same model structure as for TC, LDL-C, and HDL-C.

The analysis population included participants with available baseline and post-intervention lipid profile data. Participants were analysed according to their allocated study group. No imputation of missing outcome data was performed; analyses were conducted as complete-case analyses.

Regression coefficients are reported with 95% confidence intervals (Cls) and *p*-values. Statistical significance was set at *p* < 0.05. Given the exploratory analyses and the limited sample size, all findings were interpreted with caution. Statistical analyses were conducted in jamovi (2.7.18.0). Figures and additional visualisations were prepared in GraphPad Prism (10.2.3).

No interim analyses were planned or performed, and no formal stopping guidelines were specified because of the short duration and low-risk dietary nature of the intervention.

## 3. Results

### 3.1. Participant Flow and Recruitment

**Figure 1:**
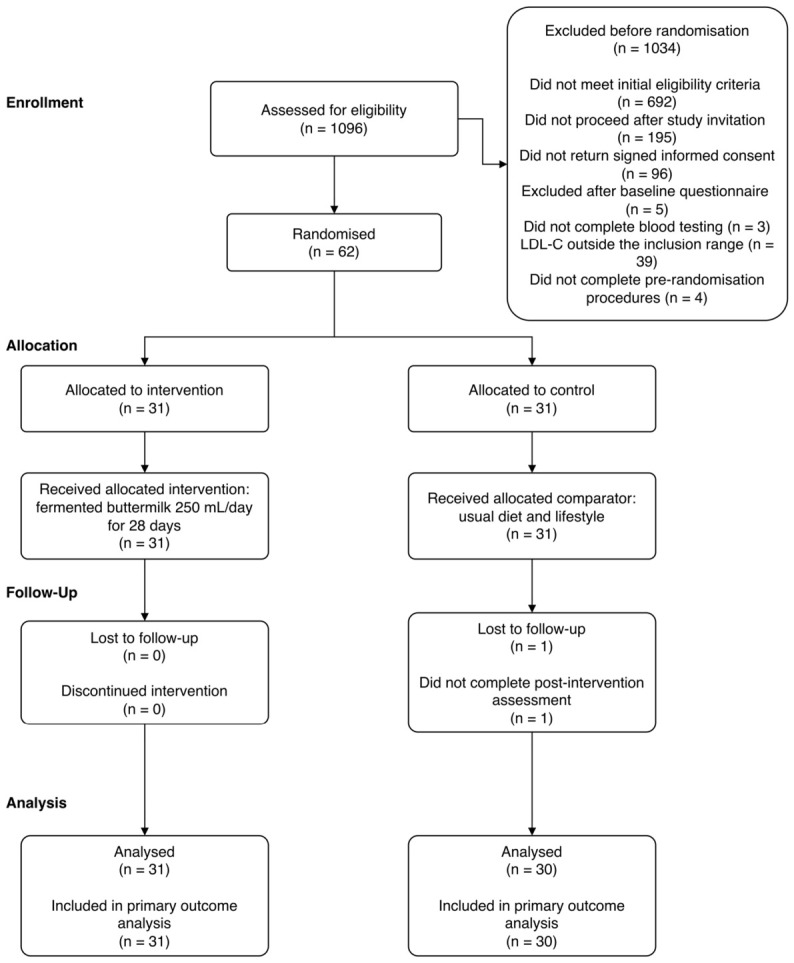
CONSORT 2025 participant flow diagram for the 28-day fermented buttermilk randomised dietary intervention.

A total of 1096 women expressed interest in participating in the study. Of these, 404 met the initial eligibility criteria and were invited to continue, and 209 expressed willingness to proceed. A total of 113 women returned signed informed consent forms. After completion of the baseline questionnaire, 108 participants remained eligible. Three participants did not complete baseline blood testing, and 39 were excluded because their LDL-C concentrations were outside the predefined inclusion range. Therefore, 66 women remained eligible after baseline testing. Of these, 62 completed the required pre-randomisation procedures and were randomised: 31 to the fermented buttermilk intervention group and 31 to the control group. One participant in the control group did not complete the post-intervention assessment. The final analysis included 61 participants: 31 in the intervention group and 30 in the control group. The CONSORT 2025 participant flow diagram is shown in Figure 1.

All participants allocated to the intervention group received the allocated fermented buttermilk intervention. The product was provided throughout the intervention period by the researcher or courier. No participant in the intervention group discontinued the intervention. Participants in the control group were instructed to maintain their usual diet and lifestyle during the same 28-day period. Quantitative adherence based on returned product containers was not assessed.

No intervention-related adverse events were reported through participant self-report during the 28-day trial period.

### 3.2. Baseline Characteristics of the Study Participants

The present secondary analysis included 61 perimenopausal women: 31 participants in the intervention group and 30 in the control group. Table 1 presents the baseline demographic, anthropometric, and lipid profile characteristics by study group and season.

Formal statistical comparisons of baseline characteristics between subgroups were not conducted because the trial was randomised, the seasonal subgroups were small, and subgroup comparisons were not part of the primary inferential analysis. Baseline characteristics are presented descriptively to provide a clinical context. To account for pre-intervention lipid status, each regression model included the corresponding baseline lipid concentration as a covariate.

Baseline, post-intervention, and change-from-baseline values for TC, LDL-C, HDL-C, and TGs are presented by study group and season in Supplementary Table S1. Descriptively, the control spring subgroup had lower post-intervention TC and LDL-C and more negative change-from-baseline values than the other subgroups, whereas values in the intervention subgroups remained closer to baseline. HDL-C and TG showed smaller, more variable changes. Because the winter and spring cohorts included different participants, these descriptive subgroup differences should not be interpreted as seasonal effects or causal intervention effects, but only as a clinical context for the adjusted regression models.

### 3.3. Chemical Composition of Dairy Products

The proximate composition of dairy products, including fat, protein, lactose, solids-not-fat, and total solids, in winter and spring is presented in Table 2.

The proximate composition of dairy products differed significantly by product type and season (all *p* < 0.05). The largest differences in fat and total solids content were observed in cream, whereas changes in skimmed milk and buttermilk were less pronounced.

Fat content showed the greatest variation between products, ranging from 0.05 to 0.07% in skimmed milk and from 38.91 to 40.51% in cream. Cream had the highest fat and total solids contents in both seasons, whereas skimmed milk had the lowest fat content. Protein, lactose, and solids-not-fat varied within narrower ranges than fat and total solids.

Several compositional parameters in raw and skimmed milk were higher in winter than in spring, whereas buttermilk fat content was higher in spring than in winter. These compositional differences are reported descriptively.

### 3.4. PL Concentrations and Relative Class Distribution in Dairy Products

Concentrations of total PL, PC, PE, and SM in the buttermilk used as the intervention product are shown in Table 3.

PL concentrations were recalculated for the 250 mL daily portion. The winter sample provided an estimated 81.30 ± 1.95 mg of total PL per daily portion, whereas the spring sample provided 69.73 ± 2.23 mg. PC and PE were the dominant PL classes in both seasonal samples. The estimated daily amounts of PC were 34.78 ± 0.75 mg in winter and 29.43 ± 0.65 mg in spring, while PE contributed 37.10 ± 1.10 mg and 36.38 ± 1.53 mg, respectively. SM contributed less to the total PL, particularly in the spring sample.

Concentrations of total PL, PC, PE, and SM in dairy products during winter and spring are shown in Table 4. Cream had the highest total PL concentration in both seasons (110.98 ± 2.69 and 110.05 ± 1.12 mg 100 g^−1^, respectively), followed by buttermilk, raw milk, and skimmed milk.

Across product-season combinations, PC and PE concentrations were higher than those of SM. Differences among PL classes were significant (*p* < 0.001), with pairwise comparisons showing differences between PC and PE (*p* = 0.034), PC and SM (*p* < 0.001), and PE and SM (*p* < 0.001).

The relative distribution of PC, PE, and SM in dairy products is shown in Table 5. Relative PC values were higher in spring than in winter for raw milk, skimmed milk, and cream, whereas buttermilk showed a slightly lower PC proportion in spring.

**Figure 2:**
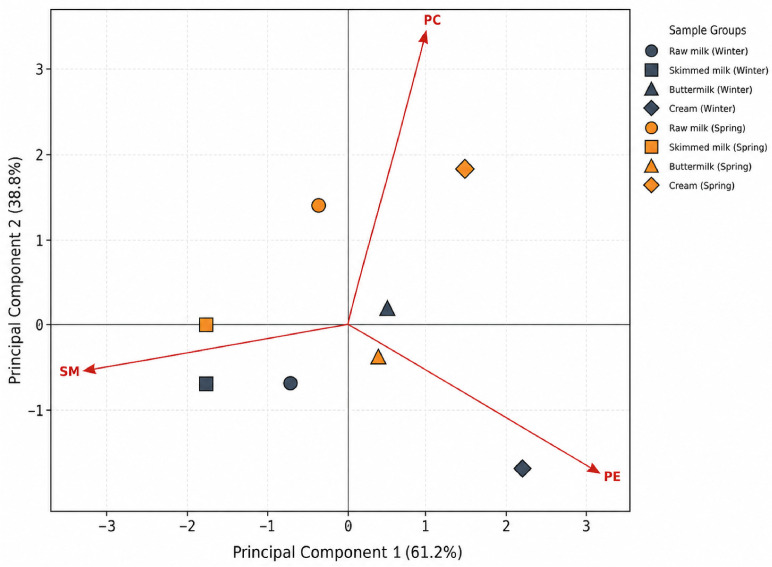
PCA biplot illustrating the multivariate structure of PL composition (PC, PE, and SM) across dairy products collected in winter and spring. Sample scores represent dairy products, while vectors indicate the PL class contributions to the principal components.

PCA of the relative PL class distribution showed that PC1 and PC2 accounted for 61.2% and 38.8% of the variance, respectively. PC1 separated samples according to the relative contributions of PE and SM, whereas PC2 was primarily aligned with PC (Table 6). The corresponding PCA biplot is shown in Figure 2.

The PCA, PC/PE ratio, and ternary plot analyses were exploratory and descriptive, given the limited number of product-season observations. They were used to characterise product-level PL composition and were not interpreted as direct evidence of participant-level lipid responses.

### 3.5. Seasonal Variations in PL Class Distribution

Seasonal variation in PL class distribution was evaluated using PC/PE ratios and compositional trajectory plots. The PC/PE ratio increased from winter to spring in raw milk, skimmed milk, and cream, with the largest change in cream (0.74 to 1.02; +37.46%). Buttermilk showed a small decrease in the PC/PE ratio (0.92 to 0.89; −4.09%) (Table 7).

**Figure 3:**
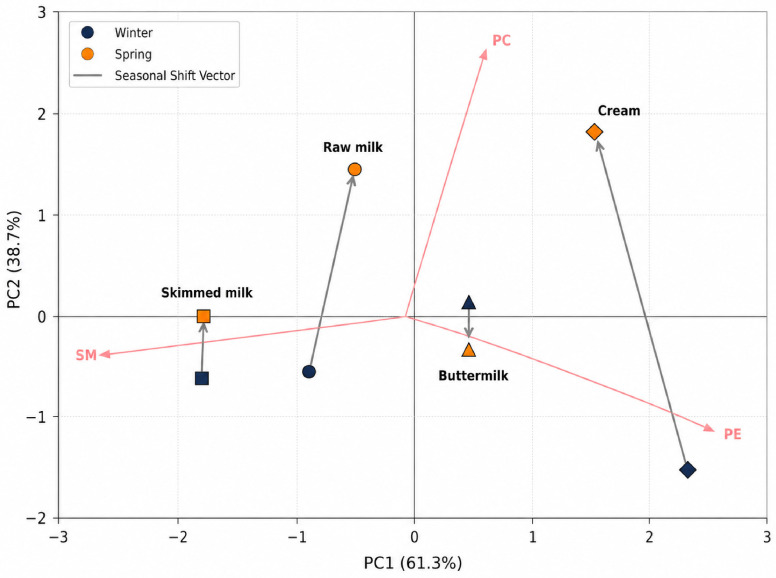
Seasonal trajectory analysis of PL class distribution in dairy products. Arrows indicate the transition from winter to spring values for each product in the PCA score space.

**Figure 4:**
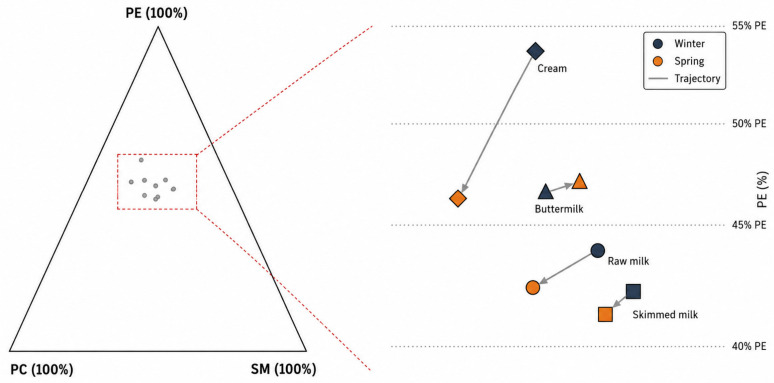
Ternary compositional trajectories of PL classes (PC, PE, and SM) in dairy products from winter to spring. Arrows represent directional shifts in relative PL class composition between seasons.

Trajectory plots visualised the direction of compositional change between winter and spring samples (Figure 3 and Figure 4). In the PCA trajectory plot, raw milk and cream shifted towards higher PC2 scores from winter to spring. In the ternary plot, raw milk, skimmed milk, and cream shifted towards a higher relative PC contribution, whereas buttermilk showed only minor displacement.

### 3.6. Estimated Dairy Product and Dairy PL Intake

Estimated intake of milk and dairy products across seasons is shown in Supplementary Table S2. Total daily intake of milk and dairy products, expressed as milk equivalents, was 947.46 g day^−1^ in winter and 894.07 g day^−1^ in spring; the seasonal difference was not statistically significant (*p* = 0.516).

The estimated total PL intake was 203.6 ± 111.7 mg day^−1^ in the intervention group and 220.2 ± 163.8 mg day^−1^ in the control group (Table 8).

Curd with 0.5% fat was the most consumed dairy product in both seasons, with intake higher in winter than in spring (193.40 vs. 104.63 g day^−1^). Semi-hard cheese intake was 104.70 g day^−1^ in winter and 113.17 g day^−1^ in spring, while soft cheese intake was 44.63 g day^−1^ in winter and 96.38 g day^−1^ in spring. Milk intake with 2.5% fat was similar between seasons (57 g day^−1^). Sweet cream with >30% fat was higher in spring, whereas butter and curd intake were higher in winter.

Estimated dairy PL intake by season is presented in Supplementary Tables S3 and S4. Based on FFQ-derived milk-equivalent intake, total dairy PL exposure was higher in winter than in spring. The summed contributions to total dairy PL intake were 264.26 mg day^−1^ in winter and 179.98 mg day^−1^ in spring. In winter, the largest contributors were curd with 0.5% fat (53.94 mg day^−1^), semi-hard cheese (29.20 mg day^−1^), butter (16.63 mg day^−1^), and milk with 2.5% fat (15.65 mg day^−1^). In spring, the largest contributors were semi-hard cheese (22.78 mg day^−1^), curd with 0.5% fat (21.06 mg day^−1^), soft cheese (19.40 mg day^−1^), and milk with 2.5% fat (11.47 mg day^−1^).

These values should be interpreted as milk-equivalent exposure estimates rather than as direct analytical PL measurements for each dairy product category consumed. PC and PE were the dominant estimated PL classes in both seasons, whereas SM contributed less to total estimated PL intake.

A two-way ANOVA of estimated absolute PL-class intake showed a main effect of season (*p* = 0.036) and of PL class (*p* < 0.001), with no significant season by PL class interaction (*p* = 0.645). This indicates that estimated PL intake differed by season and PL class, whereas the seasonal pattern did not differ significantly across PC, PE, and SM.

### 3.7. Association Between Estimated Dairy PL Exposure and Post-Intervention Plasma Lipid Outcomes

Post-intervention lipid outcome data were available for 31 participants in the intervention group and 30 participants in the control group. These participants were included in the analyses of TC, LDL-C, HDL-C, and TG. No imputation was performed for the participant who did not complete the post-intervention assessment.

Ordinary least-squares (OLS) regression models were used to evaluate post-intervention concentrations of TC, HDL-C, LDL-C, and TG, with adjustment for baseline lipid concentrations, intervention group, season, and ln(total quantified dairy PL intake). TG was included as an additional secondary lipid outcome. This participant-level analysis represents the main exposure-outcome analysis of the study; the preceding PCA, PC/PE ratio, and ternary plot analyses were used to describe the product-level PL composition. Regression results are presented in Table 9.

Baseline lipid concentrations were associated with post-intervention TC, HDL-C, LDL-C, and TG across all models (*p* < 0.01). After adjustment for baseline lipid concentrations, season, and log-transformed estimated total dairy PL intake, the intervention group allocation was associated with higher post-intervention TC (β = 0.485; 95% CI: 0.023 to 0.948; *p* < 0.05) and LDL-C (β = 0.330; 95% CI: 0.024 to 0.636; *p* < 0.05) than the control group. The coefficients for HDL-C (β = 0.099; 95% CI: −0.034 to 0.231) and TG (β = 0.052; 95% CI: −0.152 to 0.255) were not statistically significant.

The positive intervention-group coefficients for post-intervention TC and LDL-C should be interpreted with caution. These findings do not support a lipid-lowering effect of the 28-day fermented buttermilk intervention in this secondary analysis. However, they should not be interpreted as evidence that fermented buttermilk increased TC or LDL-C, as the present model was designed to evaluate post-intervention lipid outcomes while adjusting for baseline lipid concentration, season, and estimated habitual dairy PL exposure. Residual confounding by unmeasured dietary changes, habitual dietary differences, variability in adherence, regression to the mean, or other lifestyle factors may have contributed to the observed group differences. Therefore, the intervention-group findings should be interpreted as adjusted group differences rather than causal evidence of an adverse effect.

In the present winter-versus-spring comparison, the spring cohort indicator was not significantly associated with post-intervention lipid outcomes after adjustment for baseline lipid concentration, intervention group, and log-transformed estimated total dairy PL intake. Log-transformed estimated total dairy PL intake was positively associated with post-intervention HDL-C concentration (β = 0.059; 95% CI: 0.020 to 0.099; *p* < 0.01), whereas no significant associations were observed for TC, LDL-C, or TG.

Exploratory sensitivity analyses were conducted by replacing the log-transformed total quantified dairy PL intake with log-transformed intake of individual PL classes, namely PC, PE, and SM. These models showed a consistent pattern: each individual PL-class estimate was positively associated with post-intervention HDL-C, whereas no statistically significant associations were observed for TC or LDL-C. The association with HDL-C was similar for PC (β = 0.067, *p* < 0.01), PE (β = 0.066, *p* < 0.01), and SM (β = 0.077, *p* < 0.01). Because these results were qualitatively similar to the primary total PL model and did not provide evidence of a clearly distinct class-specific association, they were interpreted as supportive sensitivity analyses rather than as separate primary models.

## 4. Discussion

### 4.1. PL Profile of Dairy Products

The fermented buttermilk used as the intervention product provided approximately 81.30 ± 1.95 mg of total PL per daily portion in winter and 69.73 ± 2.23 mg in spring. These values were lower than the PL concentrations observed in the laboratory-produced buttermilk fraction, indicating that PL exposure from commercial fermented buttermilk may differ from that estimated from buttermilk fractions produced under controlled laboratory processing conditions.

Milk PLs, mainly located in the MFGM, represent a minor fraction of total milk lipids. Their amphiphilic molecular structure and membrane-associated nature may contribute to bioactive properties relevant to lipid digestion, absorption, and metabolism. Together, PE, PC, and SM account for approximately 80% of total milk PL [6,30,31]. In the present study, the relative distribution of PC, PE, and SM varied across dairy products and seasonal cohorts, consistent with previous evidence that milk PL composition varies with biological, environmental, and processing-related factors [8,32,33].

Product type was a key determinant of PL concentration and class distribution. Cream had the highest total PL concentration in both seasons, followed by buttermilk, raw milk, and skimmed milk. This pattern aligns with the association of PLs with MFGM-derived lipid fractions and the redistribution of membrane-associated lipids during separation and churning. During butter production, partial disruption of fat globules promotes the transfer of core lipids into butter, whereas MFGM fragments are preferentially retained in buttermilk [14,30,34]. Cream ripening before churning may further promote fat crystallisation, MFGM deformation or disruption, and the release of membrane-associated lipid components into the aqueous phase, including buttermilk.

Seasonal shifts in PL class balance were most evident in the PC/PE ratio, particularly in cream, where the ratio increased from 0.74 in winter to 1.02 in spring. Raw milk and skimmed milk also showed higher PC/PE ratios in spring, whereas buttermilk showed a small decrease. The PC/PE ratio is commonly used as an indicator of membrane lipid balance and may reflect differences in membrane curvature and lipid organisation [8,14,35,36]. The higher relative PC contribution in spring, especially in cream, may be linked to seasonal production factors, including feeding-related changes reported in previous studies; however, feeding regimen, lactation stage, and farm-level production variables were not measured in this study [19,37].

A recent review by Fappani et al. further supports the view that advanced LC-MS-based lipidomics can characterise the molecular diversity of bovine milk PL and that the milk polar lipidome may be shaped by animal diet, lactation stage, metabolic status, and markers such as PC/PE balance, SM, ceramide profiles, and lysophospholipid enrichment. Therefore, the present findings are consistent with the view that dairy PL composition is influenced by both biological and processing-related factors, although the current targeted method was limited to quantifying selected PC, PE, and SM species [38].

The PCA and ternary analyses provided complementary descriptive information on the distribution of the PL class. The opposing loadings of PE and SM in the PCA indicate compositional differentiation among product-season samples rather than direct evidence of structural changes in the MFGM. Similarly, the ternary trajectories showed that raw milk, skimmed milk, and cream shifted towards higher relative PC contributions in spring, whereas buttermilk showed only a minor displacement. These patterns should be interpreted as exploratory, as the dataset includes only a limited number of product-season observations. The relatively stable PE contribution across seasons is consistent with its established role in membrane curvature and flexibility, but membrane integrity was not directly assessed [14,16,32,39].

The compositional results indicate that seasonal variation in dairy PL composition was driven mainly by shifts in PC and SM, while PE remained comparatively stable. The combination of PC/PE ratio assessment and ternary visualisation provided a concise descriptive approach for examining changes in PL class balance across dairy products and seasons.

### 4.2. Seasonal Dairy Product Intake and Estimated Dairy PL Exposure

The dietary relevance of dairy PL depends on both its concentration in dairy products and the volume consumed. In this cohort, total dairy intake, expressed as milk equivalents, did not differ significantly between seasons (947.46 g day^−1^ in winter and 894.07 g day^−1^ in spring; *p* = 0.516). These values appear higher than dairy intake estimates reported in several European studies. In the EPIC calibration study, total dairy product consumption among women ranged from approximately 150 to 480 g day^−1^ across European centres [40]. Similarly, the Swiss menuCH survey reported a mean total dairy intake of 216.5 g day^−1^ [41]. In the Malmö Diet and Cancer cohort, average dairy product intakes were also lower when individual dairy categories were considered [42]. However, these comparisons should be interpreted with caution because the present study reported dairy intake as milk equivalents, whereas most European studies reported the actual weight of the product consumed.

Therefore, seasonal differences in estimated PL exposure appear to be more closely linked to product selection and product-specific PL concentrations than to total dairy product intake [6,43,44].

Seasonal consumption patterns varied across cohorts. Winter intake was characterised by higher consumption of low-fat curd (0.5% fat), butter, and several staple dairy products, whereas spring intake showed greater contributions from soft cheese, semi-hard cheese, and sweet cream (>30% fat). These patterns align with previous research indicating that dietary habits may vary seasonally with environmental, behavioural, and cultural factors [45,46,47]. Product availability, habitual food choices, and seasonal eating patterns may all contribute to these differences, although these determinants were not directly assessed.

Using product-specific mean contributions, the estimated total dairy PL intake was higher in winter than in spring. The largest contributors to winter intake were curd (0.5% fat), semi-hard cheese, butter, and milk (2.5% fat). In spring, semi-hard cheese, curd (0.5% fat), soft cheese, and milk (2.5% fat) were the main contributors. Thus, estimated dairy PL exposure was influenced not only by high-fat dairy categories, such as butter and sweet cream, but also by frequently consumed milk-equivalent dairy categories, including curd, semi-hard cheese, and milk.

A two-way ANOVA of estimated PL class intake showed a main effect of season (*p* = 0.036) and of PL class (*p* < 0.001), with no evidence of a season by PL class interaction (*p* = 0.645). This indicates that estimated intake differed by season and PL class, whereas the available data did not show a seasonal pattern across PC, PE, and SM. Across both seasonal cohorts, PC and PE were the dominant estimated PL classes, whereas SM contributed less to total estimated PL intake.

### 4.3. Association Between Estimated Dairy PL Intake and Plasma Lipid Profile

The adjusted regression models provide the primary statistical framework for interpreting estimated dairy PL exposure in relation to post-intervention lipid outcomes. Baseline lipid concentrations were the strongest predictors of post-intervention TC, HDL-C, LDL-C, and TG, as expected, because short-term dietary exposures are evaluated against an underlying lipid profile shaped by age, hormonal status, adiposity, genetics, and habitual diet. This is particularly relevant in perimenopausal women, as the menopausal transition is associated with adverse changes in lipid metabolism, including increases in TC, LDL-C, and TG, and alterations in HDL-C function and concentration. Therefore, adjustment for baseline lipid values was necessary to reduce confounding by pre-existing lipid status and to evaluate whether estimated dairy PL exposure added explanatory value beyond baseline lipid concentrations and intervention allocation [44,48,49].

The positive intervention-group coefficients for post-intervention TC and LDL-C should be interpreted cautiously. As shown in Supplementary Table S1, TC and LDL-C values in the intervention subgroups remained close to baseline, whereas the control spring subgroup showed more pronounced decreases. Thus, the higher adjusted post-intervention TC and LDL-C concentrations in the intervention group likely reflect relative between-group differences after adjustment rather than direct evidence of an adverse effect of fermented buttermilk.

Season was included in the regression models because both dietary behaviour and dairy product composition may vary across the year. Previous population-based evidence indicates that diet quality and food-group intake can vary seasonally, and FFQ-based dietary assessment is particularly sensitive to habitual intake patterns and retrospective reporting. In the present analysis, after adjustment for baseline lipid concentrations, intervention status, and log-transformed estimated total dairy PL intake, the season indicator (spring versus winter) was not significantly associated with post-intervention lipid outcomes, including TG. Therefore, the adjusted regression models did not support an independent association between seasonal cohort assignment and lipid outcomes in this sample. However, this result should not be interpreted as evidence that season is biologically irrelevant, because season may still influence estimated PL exposure indirectly through dairy product selection and season-specific PL composition [43].

This finding should be interpreted specifically as a winter-versus-spring comparison. The present study did not include summer or autumn cohorts and therefore cannot rule out the possibility that a winter-versus-summer comparison would yield different results. Seasonal contrasts in summer may be more pronounced due to potential differences in dairy production conditions, habitual dietary choices, physical activity, daylight exposure, and cardiometabolic parameters. Therefore, the absence of an independent association for the spring-versus-winter indicator should not be taken as evidence against seasonal effects across the full annual cycle.

Log-transformed total dairy PL intake was positively associated with post-intervention HDL-C concentrations, whereas no statistically significant associations were observed for TC, LDL-C or TG. Including TG as an additional secondary lipid outcome strengthens the cardiometabolic interpretation of the lipid profile. In the present analysis, estimated dairy PL exposure was associated with HDL-C but not with TG, suggesting that the observed association may be more closely related to HDL-C-related lipid transport pathways than to triglyceride metabolism. However, this finding should be interpreted with caution because the study was a secondary, exposure-based analysis and was not primarily designed to test TG-specific responses. This pattern indicates that estimated dairy PL exposure was more consistently associated with HDL-C than with cholesterol-lowering outcomes in the adjusted models. The positive association with HDL-C may reflect differences in lipid transport-related pathways rather than a direct cholesterol-lowering effect. However, recent evidence remains inconsistent, with MFGM phospholipid supplementation showing clearer effects on TC and LDL-C than on HDL-C or TG, while studies of fermented or full-fat dairy products report heterogeneous effects on HDL-C and lipoprotein-related outcomes [50,51].

Experimental and clinical studies suggest that milk PL may influence lipid metabolism through mechanisms related to intestinal cholesterol absorption, SM-cholesterol interactions, bile acid metabolism, and the MFGM matrix. However, the present study estimated habitual exposure to naturally occurring dairy PL rather than administering a standardised PL-enriched ingredient. Therefore, the observed association with HDL-C should be interpreted as an exposure-related association rather than as evidence of a direct lipid-modifying effect [13,52].

The absence of statistically significant inverse associations between estimated total dairy PL intake and TC or LDL-C contrasts with some controlled studies using milk PL-enriched ingredients, MFGM fractions, or buttermilk-derived concentrates [6,7,8,12]. Several factors may explain this discrepancy, including the use of naturally occurring PL exposure rather than enriched PL formulations, the relatively short intervention duration, the moderate sample size, and reliance on estimated habitual dairy PL intake derived from FFQ data and season-specific compositional measurements. These factors limit comparability with trials using controlled doses of purified or enriched dairy PL.

### 4.4. Strengths and Limitations

A strength of this study is the integration of experimentally quantified, season-specific dairy PL composition with participant-level dietary intake data. This approach enabled examination of estimated exposure to total dairy PL and major PL classes in relation to post-intervention plasma lipid outcomes, rather than relying solely on generic food composition values. Including dairy products at different stages of processing also improves the relevance of the compositional data for both food science and nutritional exposure assessment.

Additional strengths include the use of LC-ESI-MRM-TQ-MS/MS to quantify selected dairy PL classes and the application of multivariate and compositional visualisation tools to characterise their distribution. These methods provided detailed information on product- and season-specific PL patterns. However, PCA, PC/PE ratio assessment, and ternary plots should be treated as exploratory, given the limited number of product-season observations.

The main limitations are the small compositional dataset, the secondary exposure-based design, and reliance on FFQ-derived intake estimates. FFQs are useful for estimating habitual intake but are subject to recall bias, portion-size estimation error, and product misclassification [53]. To minimise these errors, the FFQ was completed with researcher assistance, portion-size estimation was supported by the food product and portion atlas, and reported food frequencies and quantities were cross-checked against 24 h and 72 h food diaries. Nevertheless, some misclassification among similar dairy products cannot be entirely ruled out, particularly for products with similar names, compositions, or fat content. Educational background may also influence food literacy and the ability to distinguish between similar food products; therefore, residual reporting error related to participant understanding remains possible. In addition, the seasonal cohorts were independent rather than repeated within the same participants, which limits the interpretation of seasonal differences. Farm-level feeding regimen, lactation stage, and broader production variables were not measured, so mechanisms underlying seasonal PL variation remain inferential.

A major limitation is the potential mismatch between the time frame of FFQ-derived habitual intake and the 28-day intervention outcome assessment. The FFQ captured usual intake over the preceding 12 months, whereas lipid outcomes were measured after a short intervention period. Therefore, the exposure variable represents estimated habitual background exposure rather than short-term PL intake during the intervention. Applying winter or spring product composition to year-round FFQ-derived intake may introduce exposure misclassification.

Because the original study was a pragmatic, food-based intervention, participant blinding was not feasible. Allocation was performed according to the block randomisation list as participants entered the study; however, no additional allocation concealment procedure was documented in the available study records. These factors should be considered when interpreting intervention-group differences in post-intervention lipid outcomes, particularly the higher adjusted post-intervention TC and LDL-C concentrations observed in the intervention group.

Another major limitation is that dairy PL concentrations were measured directly in a limited set of dairy fractions and products, with sampling conducted at two seasonal time points. Therefore, the compositional dataset may not capture the full variability of dairy products available in the Latvian retail sector. In addition, PL exposure across several dairy categories consumed was estimated using a milk-equivalent conversion rather than by directly quantifying each food item. This approach introduces uncertainty and may lead to misclassification of exposure, particularly for processed dairy products such as curd, cheese, butter, or sweet cream. Consequently, the reported PL intake values should be interpreted as estimates of exposure rather than precise measurements of individual PL intake.

Because the study included only winter and spring cohorts, the findings should not be interpreted as representing the full annual seasonal variation in dairy PL composition, dietary PL exposure, or lipid outcomes.

An additional limitation is that the targeted LC-ESI-MRM-TQ-MS/MS method quantified only selected dairy PL species, namely PC(16:0/18:1), PC(18:1/18:1), PE(16:0/18:2), PE(16:0/18:1), SM(d18:1/16:0), and SM(d18:1/18:0). Other milk PL classes, including PS, PI, ceramide-related sphingolipids, and other minor PL, were not included in the validated quantitative scope of the present study. Therefore, the exposure estimates reflect only the quantified PC, PE, and SM species and should not be interpreted as representing the complete composition of dairy PL.

The study population was limited to perimenopausal women in Latvia with moderately elevated LDL-C concentrations, which restricts generalisability to other populations, age groups, and cardiometabolic risk profiles. The exposure estimates included selected dairy PL classes and were based on product categories matched to measured compositional data; therefore, they may not capture the full diversity of dairy PL intake. Because the analysis was secondary and exposure-based, associations between estimated dairy PL intake and lipid outcomes should not be interpreted as causal.

## 5. Conclusions

This study highlights the importance of integrating season-specific dairy PL composition with dietary intake data when evaluating dietary exposure to bioactive lipids in perimenopausal women. The findings indicate that dairy product type and seasonal variation influence the estimated intake and relative distribution of PL classes, particularly PC, PE, and SM. Therefore, dietary exposure to dairy PL cannot be accurately inferred from total dairy intake alone and should be assessed using product-specific compositional data.

The adjusted regression models showed a positive association between estimated total dairy PL intake and HDL-C concentrations. However, estimated total dairy PL intake was not significantly associated with TC, LDL-C, or TG, and the positive point estimates for these outcomes do not support a clear lipid-lowering effect within the present study design. In the available winter and spring cohorts, the spring-versus-winter season indicator was not independently associated with lipid outcomes after adjustment for baseline lipid concentrations, intervention status, and total dairy PL intake.

The study results should be interpreted with caution, given the limited sample size, short intervention period, FFQ-based estimation of dietary exposure, and the secondary nature of the analysis. Future studies with more detailed assessments of dairy matrices are needed to further clarify the relationship between dairy PL exposure, the menopausal transition, and plasma lipid outcomes.

## Figures and Tables

**Table 1:** Baseline demographic, anthropometric, and lipid profile characteristics by study group and season.

Characteristic	Total Cohort (*n* = 61)	Intervention (*n* = 31)		Control (*n* = 30)	
		Winter (*n* = 11)	Spring (*n* = 20)	Winter (*n* = 12)	Spring (*n* = 18)
Age, years	49.39 ± 2.88	48.36 ± 2.66	49.60 ± 2.52	49.92 ± 2.97	49.44 ± 3.36
BMI, kg m^−2^	25.28 ± 4.47	25.52 ± 3.74	25.10 ± 4.44	26.58 ± 5.46	24.47 ± 4.36
Waist-to-hip ratio	0.81 ± 0.07	0.84 ± 0.03	0.80 ± 0.06	0.85 ± 0.10	0.79 ± 0.07
TC, mmol L^−1^	5.93 ± 0.54	5.86 ± 0.58	5.97 ± 0.63	5.99 ± 0.47	5.89 ± 0.50
LDL-C, mmol L^−1^	3.61 ± 0.30	3.55 ± 0.30	3.61 ± 0.32	3.66 ± 0.25	3.60 ± 0.32
HDL-C, mmol L^−1^	1.85 ± 0.46	1.77 ± 0.52	2.00 ± 0.56	1.78 ± 0.35	1.79 ± 0.34
TG, mmol L^−1^	1.15 ± 0.64	1.60 ± 0.88	0.87 ± 0.26	1.35 ± 0.70	1.05 ± 0.59

Note: Data are presented as mean ± SD. BMI, body mass index; TC, total cholesterol; LDL-C, low-density lipoprotein cholesterol; HDL-C, high-density lipoprotein cholesterol; TG, triglyceride.

**Table 2:** Seasonal proximate composition of dairy products, %.

Product	Season	Fat, %	Protein, %	Lactose, %	Solids-Not-Fat, %	Total Solids, %
Raw milk	Winter	3.83 ± 0.00	3.35 ± 0.01	4.83 ± 0.01	8.87 ± 0.02	12.69 ± 0.03
	Spring	3.81 ± 0.00	3.26 ± 0.00	4.64 ± 0.00	8.60 ± 0.01	12.41 ± 0.00
Skimmed milk	Winter	0.07 ± 0.00	3.42 ± 0.01	4.77 ± 0.01	9.09 ± 0.02	9.26 ± 0.02
	Spring	0.05 ± 0.00	3.32 ± 0.00	4.59 ± 0.01	8.80 ± 0.01	9.01 ± 0.00
Buttermilk	Winter	0.78 ± 0.00	2.90 ± 0.01	4.53 ± 0.01	8.27 ± 0.01	9.16 ± 0.01
	Spring	0.93 ± 0.00	2.83 ± 0.04	4.50 ± 0.00	8.24 ± 0.00	9.12 ± 0.00
Cream	Winter	40.51 ± 0.04	1.99 ± 0.00	2.75 ± 0.01	5.62 ± 0.05	46.14 ± 0.06
	Spring	38.91 ± 0.07	2.01 ± 0.01	2.73 ± 0.01	5.59 ± 0.01	44.56 ± 0.08

Note: Data are presented as mean ± SD.

**Table 3:** PL concentrations and estimated daily PL amount supplied by the fermented buttermilk used as the intervention product.

PL Class	Winter		Spring	
	mg 100 g^−1^	mg per 250 mL	mg 100 g^−1^	mg per 250 mL
Total PL	32.52 ± 0.78	81.30 ± 1.95	27.89 ± 0.89	69.73 ± 2.23
PC	13.91 ± 0.30	34.78 ± 0.75	11.77 ± 0.26	29.43 ± 0.65
PE	14.84 ± 0.44	37.10 ± 1.10	14.55 ± 0.61	36.38 ± 1.53
SM	3.77 ± 0.05	9.43 ± 0.13	1.57 ± 0.05	3.93 ± 0.13

Note: Data are presented as mean ± SD. The estimated amount per 250 mL was calculated assuming 250 mL ≈ 250 g of product.

**Table 4:** PL concentrations in dairy products (raw milk, skimmed milk, buttermilk, and cream are produced in the laboratory in winter and spring).

Product	Season	Total PL, mg 100 g^−1^	PC, mg 100 g^−1^	PE, mg 100 g^−1^	SM, mg 100 g^−1^
Raw milk	Winter	27.89 ± 1.93	11.23 ± 0.5	12.42 ± 0.7	4.24 ± 0.2
	Spring	20.13 ± 0.14	9.04 ± 0.1	8.52 ± 0.1	2.57 ± 0.1
Skimmed milk	Winter	11.97 ± 0.61	4.76 ± 0.2	5.04 ± 0.2	2.17 ± 0.1
	Spring	10.83 ± 1.52	4.47 ± 0.4	4.45 ± 0.3	1.91 ± 0.2
Buttermilk	Winter	51.65 ± 0.36	22.07 ± 0.1	23.87 ± 0.1	5.71 ± 0.1
	Spring	44.51 ± 1.99	18.51 ± 0.1	20.88 ± 0.8	5.11 ± 0.4
Cream	Winter	110.98 ± 2.69	43.95 ± 0.3	59.35 ± 1.2	7.68 ± 0.4
	Spring	110.05 ± 1.12	51.44 ± 0.5	50.53 ± 0.2	8.08 ± 0.1

Note: Data are presented as mean ± SD, mg 100 g^−1^.

**Table 5:** Relative PL class distribution in dairy products in winter and spring, %.

Season	Product	PC, %	PE, %	SM, %
Winter	Raw milk	40.30	44.50	15.20
	Skimmed milk	39.80	42.20	18.10
	Buttermilk	42.70	46.20	11.10
	Cream	39.60	53.50	6.90
Spring	Raw milk	44.90	42.30	12.80
	Skimmed milk	41.20	41.10	17.60
	Buttermilk	41.59	46.92	11.49
	Cream	46.70	45.90	7.30

**Table 6:** Loadings of PL variables on the first two principal components derived from PCA of standardised compositional data.

Variable	PC1 (61.2%)	PC2 (38.8%)
PC	0.304	1.025
PE	0.943	−0.504
SM	−1.058	−0.155

Note: PC1, first principal component; PC2, second principal component.

**Table 7:** Seasonal variation in the PC/PE ratio across dairy products.

Product	Winter	Spring	Change (%)
Raw milk	0.91	1.06	+16.99%
Skimmed milk	0.94	1.00	+6.29%
Buttermilk	0.92	0.89	−4.09%
Cream	0.74	1.02	+37.46%

**Table 8:** Participant distribution across seasonal cohorts and estimated total quantified dairy PL intake by the study group.

	Intervention Group	Control Group
Seasonal cohort		
Winter	11 (35.5%)	12 (40%)
Spring	20 (64.5%)	18 (60%)
Estimated total quantified dairy PL intake, mg day^−1^		
Mean	203.6	220.2
SD	111.7	163.8
Median	217.3	196.8

Note: Data are presented as *n* (%) for seasonal cohort membership and as mean, SD, and median for estimated total quantified dairy PL intake. Percentages are calculated within each study group. The estimated total quantified dairy PL intake is expressed as mg day^−1^ and defined as the sum of quantified PC, PE, and SM.

**Table 9:** Associations between estimated dairy PL intake and post-intervention plasma lipid concentrations.

	TC After Intervention	HDL-C After Intervention	LDL-C After Intervention	TG After Intervention
Baseline	0.752 **	0.769 **	0.750 **	0.561 **
	[0.532 0.973]	[0.653 0.885]	[0.448 1.052]	[0.385 0.736]
Intervention	0.485 *	0.099	0.330 *	0.052
	[0.023 0.948]	[−0.034 0.231]	[0.024 0.636]	[−0.152 0.255]
Spring	−0.183	−0.016	−0.091	−0.098
	[−0.665 0.298]	[−0.151 0.118]	[−0.417 0.235]	[−0.340 0.145]
log(PL)	0.206	0.059 **	0.130	−0.034
	[−0.030 0.442]	[0.020 0.099]	[−0.067 0.327]	[−0.183 0.114]
Number of observations	61	61	61	61

* *p* < 0.05; ** *p* < 0.01. Note: Values are regression coefficients with 95% confidence intervals in square brackets. Models were adjusted for baseline lipid concentration, intervention group, season, and log-transformed total dairy PL intake.

## Data Availability

The de-identified data supporting the findings of this study are available from the corresponding author upon reasonable request and subject to ethical and privacy restrictions. The data are not publicly available because they contain information that could compromise participant privacy. Statistical code and additional analysis materials are available from the corresponding author upon reasonable request.

## Supplementary Materials

The following supporting information can be downloaded at: https://www.mdpi.com/article/10.3390/nu18132194/s1, Table S1. Baseline, post-intervention, and change in lipid outcomes by study group and season; Table S2. Average daily intake of milk and dairy products expressed as milk equivalents in the pooled winter (*n* = 23) and spring (*n* = 38) seasonal cohorts; Table S3. The estimated average daily intake of dairy PL during winter, based on pooled winter cohort FFQ-derived dairy intake (*n* = 23); Table S4. The estimated average daily intake of dairy PL during the spring, based on pooled spring cohort FFQ-derived dairy intake (*n* = 38); Table S5. MRM transitions and retention times used for targeted LC-ESI-MRM-TQ-MS/MS analysis of PL classes; Figure S1. Extracted ion chromatograms (EICs) of selected phospholipid standards obtained by LC-ESI-MRM-TQ-MS/MS.
