# Supplementary material for: Estimated Habitual Dairy Polar Lipid Exposure and Post-Intervention Plasma Lipid Outcomes in Perimenopausal Women in Latvia: A Secondary Exposure-Based Analysis of a 28-Day Fermented Buttermilk Randomised Trial
Source: Nutrients. 2026 Jul 6;18(13):2194. doi: 10.3390/nu18132194 (PMC13363648; doi:10.3390/nu18132194)
Supplement: Supplementary file 1 [file nutrients-18-02194-s001.zip › nutrients-4371264-supplementary.pdf]

Supplementary materials

Table S1. Baseline, post-intervention, and change in lipid outcomes by study group and season.

Lipid outcome	Total cohort (n = 61)	Intervention winter (n = 11)	Intervention spring (n = 20)	Control winter (n = 12)	Control spring (n = 18)
TC baseline, mmol L^{-1}	5.93 ± 0.54	5.86 ± 0.58	5.97 ± 0.63	5.99 ± 0.47	5.89 ± 0.50
TC after 28 days, mmol L^{-1}	5.66 ± 0.95	5.83 ± 0.57	5.91 ± 0.88	5.84 ± 1.22	5.17 ± 0.90
TC change, mmol L^{-1}	-0.27 ± 0.95	-0.03 ± 0.70	-0.06 ± 0.80	-0.15 ± 1.21	-0.73 ± 0.96
LDL-C baseline, mmol L^{-1}	3.61 ± 0.30	3.55 ± 0.30	3.61 ± 0.32	3.66 ± 0.25	3.60 ± 0.32
LDL-C after 28 days, mmol L^{-1}	3.49 ± 0.65	3.64 ± 0.51	3.62 ± 0.67	3.51 ± 0.73	3.23 ± 0.60
LDL-C change, mmol L^{-1}	-0.12 ± 0.62	0.09 ± 0.40	0.01 ± 0.61	-0.14 ± 0.79	-0.38 ± 0.57
HDL-C baseline, mmol L^{-1}	1.85 ± 0.46	1.77 ± 0.52	2.00 ± 0.56	1.78 ± 0.35	1.79 ± 0.34
HDL-C after 28 days, mmol L^{-1}	1.77 ± 0.42	1.77 ± 0.47	1.92 ± 0.43	1.71 ± 0.45	1.64 ± 0.35
HDL-C change, mmol L^{-1}	-0.08 ± 0.28	-0.00 ± 0.21	-0.08 ± 0.29	-0.07 ± 0.32	-0.14 ± 0.29
TG baseline, mmol L^{-1}	1.15 ± 0.64	1.60 ± 0.88	0.87 ± 0.26	1.35 ± 0.70	1.05 ± 0.59
TG after 28 days, mmol L^{-1}	1.09 ± 0.54	1.31 ± 0.61	1.00 ± 0.34	1.33 ± 0.77	0.91 ± 0.42
TG change, mmol L^{-1}	-0.06 ± 0.47	-0.29 ± 0.71	0.12 ± 0.27	-0.02 ± 0.43	-0.14 ± 0.43

Note: Data are presented as mean ± SD. Change was calculated as after 28 days minus baseline for each participant. TC, total cholesterol; LDL-C, low-density lipoprotein cholesterol; HDL-C, high-density lipoprotein cholesterol; TG, triglycerides.

Table S2. Average daily intake of milk and dairy products expressed as milk equivalents in the pooled winter (n = 23) and spring (n = 38) seasonal cohorts.

Products	Winter (g day^{-1})	Spring (g day^{-1})
Curd cream	42.99	14.47
Glazed curd snack (with chocolate coating)	29.81	22.80
Curd 0.5% fat	193.40	104.63
Curd 5% fat	53.53	33.41
Curd 9% fat	21.80	65.10
Fermented milk products (kefir, yogurt, ayran) without additives <2% fat	35.05	40.10
Fermented milk products (kefir, yogurt, cultured milk, ayran) without additives 2.1–5% fat	36.30	30.21
Fermented milk products (kefir, yogurt, cultured milk) with additives 2.1–5% fat	12.57	7.39
Fermented milk products (kefir, yogurt) with additives <2% fat	9.29	6.90
Ghee (clarified butter)	5.29	9.04
Cream <20% fat	37.03	33.02
Cream >25% fat	7.97	13.37
Soft cheese	44.63	96.38
Buttermilk with additives <1% fat	4.62	2.31
Buttermilk with additives >1.5% fat	4.17	2.36
Buttermilk without additives <1% fat	15.81	17.52
Buttermilk without additives >1.5% fat	9.59	10.06
High-protein dairy products ("Piena spēks", skyr, etc.) with additives	6.08	2.70
High-protein dairy products ("Piena spēks", skyr, etc.) without additives	21.08	22.04
Milk 0.5% fat	12.54	2.45
Milk 1.5% fat	0.02	0.76
Milk 2.5% fat	56.10	57.00
Milk 3.2% fat	9.50	6.92
Flavored milk	1.29	0.03
Sweet cream <15% fat	12.84	8.38
Sweet cream >30% fat	14.20	36.94
Hard cheese (e.g., Parmesan)	36.63	47.54
Semi-hard cheese	104.70	113.17
Fresh cheese / cream cheese (e.g., Philadelphia, Kārums)	49.00	52.22
Butter	59.63	34.85

Notes: Values represent cohort-level average estimates calculated for all participants assessed in the corresponding season, with intervention and control participants pooled. This table describes seasonal dietary exposure profiles and is not stratified by intervention group.

Table S3. The estimated average daily intake of dairy PL during winter, based on pooled winter cohort FFQ-derived dairy intake (n = 23).

Products	Total PL, mg day^{-1}	PC, mg day^{-1}	PE, mg day^{-1}	SM, mg day^{-1}
Curd cream	11.99	4.83	5.34	1.82
Glazed curd snack (with chocolate coating)	8.32	3.35	3.70	1.26
Curd 0.5% fat	53.94	21.74	24.00	8.20
Curd 5% fat	14.93	6.02	6.64	2.27
Curd 9% fat	6.08	2.45	2.71	0.92
Fermented milk products (kefir, yogurt, ayran) without additives <2% fat	9.78	3.94	4.35	1.49
Fermented milk products (kefir, yogurt, cultured milk, ayran) without additives 2.1–5% fat	10.12	4.08	4.51	1.54
Fermented milk products (kefir, yogurt, cultured milk) with additives 2.1–5% fat	3.50	1.41	1.56	0.53
Fermented milk products (kefir, yogurt) with additives <2% fat	2.59	1.04	1.15	0.39
Ghee (clarified butter)	1.48	0.59	0.66	0.22
Cream <20% fat	10.33	4.16	4.60	1.57
Cream >25% fat	2.22	0.90	0.99	0.34
Soft cheese	12.45	5.02	5.54	1.89
Buttermilk with additives <1% fat	1.29	0.52	0.57	0.20
Buttermilk with additives >1.5% fat	1.16	0.47	0.52	0.18
Buttermilk without additives <1% fat	4.41	1.78	1.96	0.67
Buttermilk without additives >1.5% fat	2.67	1.08	1.19	0.41
High-protein dairy products ("Piena spēks", skyr, etc.) with additives	1.69	0.68	0.75	0.26
High-protein dairy products ("Piena spēks", skyr, etc.) without additives	5.88	2.37	2.62	0.89
Milk 0.5% fat	3.50	1.41	1.56	0.53
Milk 1.5% fat	0.01	0.00	0.00	0.00
Milk 2.5% fat	15.65	6.31	6.96	2.38
Milk 3.2% fat	2.65	1.07	1.18	0.40
Flavored milk	0.36	0.14	0.16	0.05
Sweet cream <15% fat	3.58	1.44	1.59	0.54
Sweet cream >30% fat	3.96	1.60	1.76	0.60
Hard cheese (e.g., Parmesan)	10.22	4.12	4.55	1.55
Semi-hard cheese	29.20	11.77	12.99	4.44
Fresh cheese / cream cheese (e.g., Philadelphia, Kārums)	13.67	5.51	6.08	2.08
Butter	16.63	6.70	7.40	2.53

Note: Values represent cohort-level average estimates calculated for all participants assessed in the corresponding season, with intervention and control participants pooled. Estimated dairy PL contributions were calculated from FFQ-derived dairy intake

expressed as milk equivalents and season-specific PL concentrations measured in raw milk, skimmed milk, cream, and buttermilk. PL concentrations were not measured directly for all listed dairy food categories; therefore, product-level values should be interpreted as milk-equivalent exposure estimates, not as analytically measured PL concentrations for each individual food item.

Table S4. The estimated average daily intake of dairy PL during the spring, based on pooled spring cohort FFQ-derived dairy intake (n = 38).

Products	Total PL, mg day^{-1}	PC, mg day^{-1}	PE, mg day^{-1}	SM, mg day^{-1}
Curd cream	2.91	1.31	1.23	0.37
Glazed curd snack (with chocolate coating)	4.59	2.06	1.94	0.59
Curd 0.5% fat	21.06	9.46	8.91	2.70
Curd 5% fat	6.73	3.02	2.84	0.86
Curd 9% fat	13.11	5.88	5.54	1.68
Fermented milk products (kefir, yogurt, ayran) without additives <2% fat	8.07	3.62	3.41	1.03
Fermented milk products (kefir, yogurt, cultured milk, ayran) without additives 2.1–5% fat	6.08	2.73	2.57	0.78
Fermented milk products (kefir, yogurt, cultured milk) with additives 2.1–5% fat	1.49	0.67	0.63	0.19
Fermented milk products (kefir, yogurt) with additives <2% fat	1.39	0.62	0.59	0.18
Ghee (clarified butter)	1.82	0.82	0.77	0.23
Cream <20% fat	6.65	2.98	2.81	0.85
Cream >25% fat	2.69	1.21	1.14	0.34
Soft cheese	19.40	8.71	8.21	2.48
Buttermilk with additives <1% fat	0.46	0.21	0.20	0.06
Buttermilk with additives >1.5% fat	0.48	0.21	0.20	0.06
Buttermilk without additives <1% fat	3.53	1.58	1.49	0.45
Buttermilk without additives >1.5% fat	2.02	0.91	0.86	0.26
High-protein dairy products ("Piena spēks", skyr, etc.) with additives	0.54	0.24	0.23	0.07
High-protein dairy products ("Piena spēks", skyr, etc.) without additives	4.44	1.99	1.88	0.57
Milk 0.5% fat	0.49	0.22	0.21	0.06
Milk 1.5% fat	0.15	0.07	0.07	0.02
Milk 2.5% fat	11.47	5.15	4.85	1.47
Milk 3.2% fat	1.39	0.63	0.59	0.18
Flavored milk	0.01	0.00	0.00	0.00
Sweet cream <15% fat	1.69	0.76	0.71	0.22
Sweet cream >30% fat	7.44	3.34	3.15	0.95
Hard cheese (e.g., Parmesan)	9.57	4.30	4.05	1.22
Semi-hard cheese	22.78	10.23	9.64	2.92

Fresh cheese / cream cheese (e.g., Philadelphia, Kārums)	10.51	4.72	4.45	1.35
Butter	7.02	3.15	2.97	0.90

Note: Values represent cohort-level average estimates calculated for all participants assessed in the corresponding season, with intervention and control participants pooled. Estimated dairy PL contributions were calculated from FFQ-derived dairy intake expressed as milk equivalents and season-specific PL concentrations measured in raw milk, skimmed milk, cream, and buttermilk. PL concentrations were not measured directly for all listed dairy food categories; therefore, product-level values should be interpreted as milk-equivalent exposure estimates, not as analytically measured PL concentrations for each individual food item.

Table S5. MRM transitions and retention times used for targeted LC-ESI-MRM-TQ-MS/MS analysis of PL classes.

Lipid species	Precursor ion, m/z	Product ion, m/z	RT, min
SM(d18:1/16:0)	704.4	184.1	14.23
SM(d18:1/18:0)	731.7	184.1	14.75
PE(16:0/18:2)	716.6	575.5	14.68
PE(16:0/18:1)	718.5	577.5	14.93
PC(16:0/18:1)	760.5	184.2	15.75
PC(18:1/18:1)	786.5	184.2	16.03

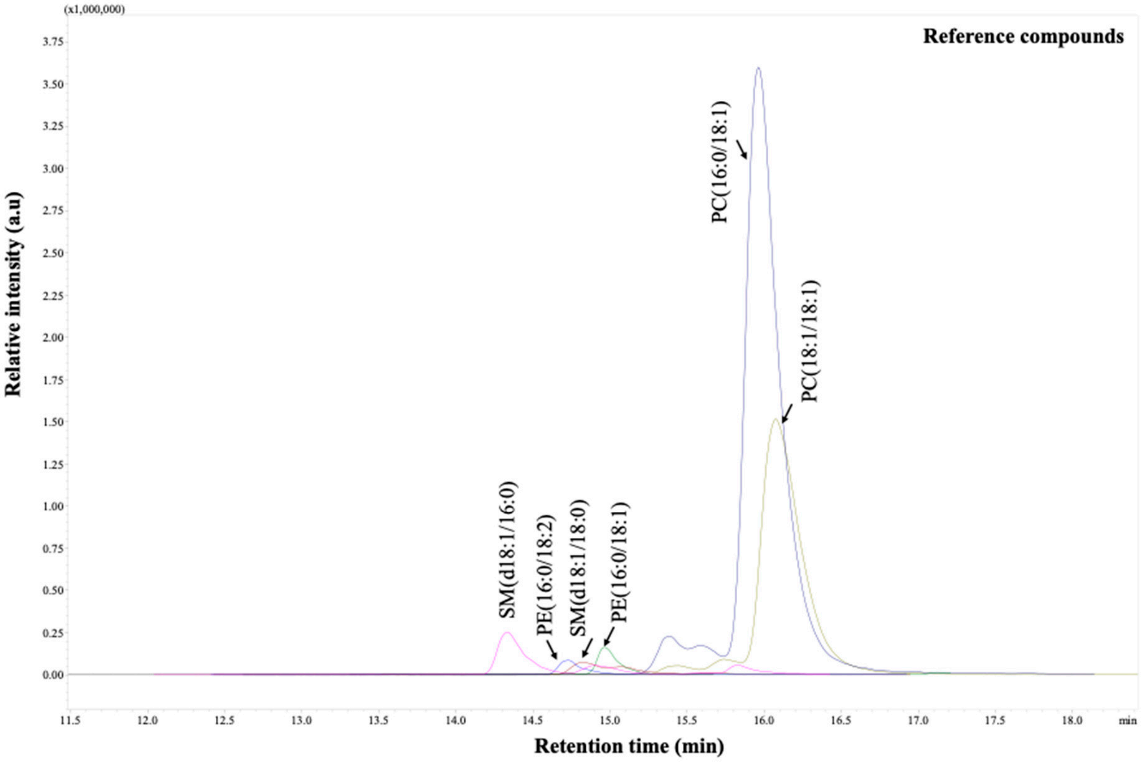

Figure S1. Extracted ion chromatograms (EICs) of selected phospholipid standards obtained by LC-ESI-MRM-TQ-MS/MS. **Note:** The chromatogram shows simultaneous detection of the targeted milk phospholipid and sphingomyelin species, including phosphatidylcholine PC(16:0/18:1) and PC(18:1/18:1), phosphatidylethanolamine PE(16:0/18:2) and PE(16:0/18:1), and sphingomyelin SM(d18:1/16:0) and SM(d18:1/18:0).
